# MicroRNAs Regulate Human Adipocyte Lipolysis: Effects of miR-145 Are Linked to TNF-α

**DOI:** 10.1371/journal.pone.0086800

**Published:** 2014-01-24

**Authors:** Silvia Lorente-Cebrián, Niklas Mejhert, Agné Kulyté, Jurga Laurencikiene, Gaby Åström, Pér Hedén, Mikael Rydén, Peter Arner

**Affiliations:** 1 Lipid Laboratory, Department of Medicine (H7) Huddinge, Karolinska Institute, Stockholm, Sweden; 2 Akademikliniken, Stockholm, Sweden; GDC, Germany

## Abstract

MicroRNAs (miRNAs) are small non-coding RNAs that regulate gene expression and have multiple effects in various tissues including adipose inflammation, a condition characterized by increased local release of the pro-lipolytic cytokine tumor necrosis factor-alpha (TNF-α). Whether miRNAs regulate adipocyte lipolysis is unknown. We set out to determine whether miRNAs affect adipocyte lipolysis in human fat cells. To this end, eleven miRNAs known to be present in human adipose tissue were over-expressed in human *in vitro* differentiated adipocytes followed by assessments of TNF-α and glycerol levels in conditioned media after 48 h. Three miRNAs (miR-145, -26a and let-7d) modulated both parameters in parallel. However, while miR-26a and let-7d decreased, miR-145 increased both glycerol release and TNF-α secretion. Further studies were focused therefore on miR-145 since this was the only stimulator of lipolysis and TNF-α secretion. Time-course analysis demonstrated that miR-145 over-expression up-regulated TNF-α expression/secretion followed by increased glycerol release. Increase in TNF-α production by miR-145 was mediated via activation of p65, a member of the NF-κB complex. In addition, miR-145 down-regulated the expression of the protease ADAM17, resulting in an increased fraction of membrane bound TNF-α, which is the more biologically active form of TNF-α. MiR-145 overexpression also increased the phosphorylation of activating serine residues in hormone sensitive lipase and decreased the mRNA expression of phosphodiesterase 3B, effects which are also observed upon TNF-α treatment in human adipocytes. We conclude that miR-145 regulates adipocyte lipolysis via multiple mechanisms involving increased production and processing of TNF-α in fat cells.

## Introduction

Obesity and insulin resistance are characterized by several disturbances in white adipose tissue (WAT) function including increased basal (i.e. non-hormone stimulated) lipolysis and a chronic low-grade inflammation. The latter results in an increased release of pro-inflammatory factors including interleukin-6 (IL-6), chemo-attractant protein chemokine (C-C motif) ligand 2 (CCL2, also known as MCP-1) and tumour necrosis factor-alpha (TNF-α) which can be produced by both adipocytes and infiltrating leucocytes (e.g. macrophages) (see [1] for review). Among these, TNF-α has gained considerable interest due to its multiple actions on adipocyte function including increased basal lipolysis and reduced insulin sensitivity which together result in a pernicious metabolic profile (reviewed in [2]).

In adipocytes, TNF-α affects lipolysis via multiple mechanisms mediated via its cognate receptor TNF-α-receptor-1 (TNFR1) [3] which in turn activate two main intracellular pathways: the mitogen activated protein kinases (MAPKs) (involving activation of ERK1/2 and JNK but not p38) [3], [4], [5] and NF-κB [6]. This results in increased phosphorylation and attenuated gene expression of perilipin-1 (PLIN1), a lipid droplet coating phosphoprotein that controls triglyceride hydrolysis by regulating access of hormone sensitive-lipase (HSL) to the lipid droplet surface [7]. TNF-α also affects HSL activity more directly by increasing protein phosphorylation at the activating residues p-Ser552, p-Ser649 and p-Ser650 and reducing it at the inactivating site p-Ser554 [8]. Furthermore, TNF-α down-regulates phosphodiesterase 3B (PDE3B), the enzyme that catalyzes cAMP hydrolysis and which mediates the antilipolytic effect of insulin [9].

The regulation of TNF-α production and secretion is complex and involves an extensive cross-talk at the intra- and extracellular level, including a self-regulatory loop [10], [11], [12]. TNF-α is synthesized as a 26-kDa trans-membrane protein which is cleaved by ADAM17, a member of the metalloproteinase family [13]. This protein cleavage results in the release of the secreted 17-kDa form of TNF-α from fat cells [14]. Although both forms of TNF-α (i.e. secreted and membrane bound) are biologically active, studies have shown that they have overlapping as well as differential biological roles (reviewed in [15]).

MicroRNAs (miRNAs) are small non-coding RNAs that regulate gene expression at the post-transcriptional level [16]. These molecules influence numerous cellular processes including adipocyte function [17]. Recent studies have demonstrated that miRNAs play an important role in the regulation of glucose metabolism, adipogenesis and inflammation in adipose tissue [18], [19], [20]. Interestingly, in non-adipose tissues several miRNAs have also been shown to control TNF-α production, for instance by regulating the expression of ADAM17 [21]. However, whether miRNAs regulate adipocyte lipolysis and production of TNF-α is not known. In this work, we screened eleven miRNAs previously shown to be considerably present in WAT of a large number of subjects [18] for their possible effects on TNF-α release and lipolysis in human primary adipocytes. Our primary aim was to identify miRNAs that could affect basal lipolysis primarily via changes in TNF production/secretion.

## Materials and Methods

### Cell Culture

Experimental (*in vitro*) studies were carried out in subcutaneous WAT obtained from healthy men and women undergoing liposuction for cosmetic reasons. None of the subjects was on any regular medication and there was no selection for age, sex or body mass index (BMI). Isolation, culture and *in vitro* differentiation of human adipocyte progenitor cells from subcutaneous WAT were performed as described previously [22]. Briefly, subcutaneous WAT was washed, cut into small pieces and digested with collagenase for 1 h at 37°C. The obtained cell suspension was centrifuged at 200×*g* for 10 min and the supernatant (containing mature adipocytes and collagenase solution), was removed. The stroma-vascular fraction (containing pre-adipocytes) was re-suspended in erythrocyte lysis buffer for 10 min, filtered through a nylon mesh and centrifuged as described above. The supernatant was discarded and the cell pellet was re-suspended in an inoculation DMEM/F12 medium supplemented with 10% fetal bovine serum, 100 µg/mL penicillin-streptomycin and was subsequently filtered through a 70 µm pore size filter. Cells were plated at the density of 30.000–50.000 cells/cm^2^ in inoculation medium to allow cells attachment. After 24 h, the medium was changed to differentiation medium (DMEM/F12 supplemented with 15 mM HEPES, 100 µg/ml penicillin-streptomycin, 2.5 µg/ml amphotericin B, 66 nM human insulin, 1 nM triiodo-L-thyroine, 10 µg/ml human transferin, 33 µM biotin, 17 µM panthotenate, 100 nM cortisol and 10 µM rosiglitazone (BRL49653). Rosiglitazone was included first 3–6 days and then removed from the differentiation medium. *In vitro* differentiated adipocytes obtained from different subjects were not pooled. Each experiment was repeated in cells isolated from at least three separate individuals. All subjects were informed about this study and written consent was obtained. This study was approved by the Research Ethics Committee at Karolinska Institutet Huddinge (Sweden).

### MicroRNA and siRNA Transfection

*In vitro* differentiated adipocytes were treated with 40 nM of miRIDIAN miRNA Mimics (Thermo Fisher Scientific, Lafayette, CO) and HiperFect Transfection Reagent (Qiagen, Hilden, Germany) at day 10–12 of differentiation according to the manufacturers’ protocols. After appropriate incubation times (6–12–24–48 h), cells were harvested for RNA or protein analysis and conditioned media was collected. Optimal transfection conditions were determined in separate titration experiments and transfection efficiency was assessed with quantitative real-time PCR (qRT-PCR) using miRNA probes (Applied Biosystems, Foster City, CA). All experiments showed at least, 100–200 fold-change up-regulation of the transfected miRNA. We did not observe any morphological change on adipocyte phenotype and transfected adipocytes remained functional 48 h post-transfection (values not shown). To rule out unspecific effects, control cells were transfected with miRIDIAN miRNA Mimic Negative Controls (Thermo Fisher Scientific).

For RNAi experiments, human differentiated adipocytes were transfected as described above with ON-TARGETplus SMARTpool siRNA against TNFR1 (Thermo Fisher Scientific) and appropriate negative control for 24 h prior to co-transfection with miRIDIAN miRNA Mimics (Neg. Cntl/miR-145) for additional 48 h.

### Lipolysis

Glycerol release into cell culture medium was determined as an index of lipolysis using a bioluminescence method as described [3] and/or using Free Glycerol Reagent (Sigma Aldrich, St. Louis, MO) and Amplex UltraRed® (Invitrogen, Carlsbad, CA) according to manufacturer instructions. Amplex Ultra Red was diluted 100-fold in Free Glycerol Reagent, mixed with 20 µL of conditioned medium in 96-well plate and incubated at room temperature for 15 min (protected from light). After incubation period (15 mins), fluorescence was measured (ex/em 530/590) using Infinite M200 plate reader (Tecan Group Ltd., Männedorf, Switzerland). Both methods yield similar results.

### RNA Isolation, cDNA Synthesis and qRT-PCR

Cells were harvested in QIAzol Lysis Reagent (Qiagen) and total RNA was isolated using miRNeasy Mini Kit (Qiagen) according to manufacturer instructions. RNA concentration as well as purity was measured spectrophotometrically using a Nanodrop ND-2000 Spectrophotometer (Thermo Fisher Scientific) and high quality RNA was confirmed using the Agilent 2100 Bioanalyzer (Agilent Technologies, Palo Alto, CA). Reverse transcription and qRT-PCR were performed as described [18]. Relative gene expression calculated using the comparative Ct-method, i.e. 2^ΔCt-target gene^/2^ΔCt-reference gene^ with *18S* and/or *LRP10* as internal control. For miRNA expression experiments (transfection efficiency), miRNA expression was normalized to reference gene *RNU48* [18].

### Enzyme-linked Immunosorbent Assay (ELISA)

TNF-α secretion levels were determined in conditioned media from *in vitro* differentiated adipocytes by ELISA according to manufacturer’s instructions (R&D Systems, Minneapolis, MN).

### NF-κB Nuclear Translocation Assay

To determine NF-κB activation by miR-145, we used TransAM NF-κB p65 kit from Active Motif (Tokyo, Japan). After appropriate transfection time with mimics of miR-145, *in vitro* differentiated adipocytes were harvested and nuclear extracts were prepared as described previously [23] with some modifications. Briefly, adipocytes were harvested and washed in PBS, resuspended to 1×10^6^ cells/mL in solution A [10 mM NaCl, 10 mM Tris-HCl (pH 7.5), 3 mM MgCl_2_, 0.05% NP-40, 1 mM PMSF, 5 mM NaF, 1 mM Na_3_VO_4_ and protease inhibitor cocktail set V, EDTA-free (Calbiochem, San Diego, CA)], and then allowed to swell for 15 min at 0°C. Thereafter, the cell suspension was shaken vigorously and mixed immediately to 1∶1 (vol/vol) with solution B (solution A supplemented with 0.6 M sucrose). The nuclei were pelleted by centrifugation at 14.000 rpm for 90 sec. Nuclei were examined under a light microscope for purity and integrity using TrypanBlue/NaCl (150 mM) solution. Nuclei from 1×10^6^ cells were subsequently re-suspended in 40–60 µL of nuclei lysis buffer (provided in TransAM NF-κB p65 kit, Active Motif) supplemented with 5 mM NaF, 1 mM Na_3_VO_4_, protease inhibitor cocktail and benzonase (Sigma). Concentration of nuclear proteins was measured using BC/RC kit (Bio-Rad, Hercules, CA). Translocation assay was performed according to manufacturer instructions. Nuclear extracts (2.5 µg) were used in binding reactions and provided Jurkat nuclear extract (1.25 µg) was used as positive control in translocation assay. Wild-type and mutated consensus oligonucleotides were used in the assay as competitors of NF-κB binding in order to assure specificity of the assay. Wild-type (but not mutated) oligonucleotides inhibited NF-κB p65 activation.

### Western Blot

Cells were lysed in RIPA buffer as described previously [24] and 15–20 µg of total protein was separated by SDS-PAGE. Western blot was performed according to standard procedures. The membranes were blocked in 3% ECL Advance™ Blocking Agent (GE Healthcare, Buckinghamshire, UK). Primary antibodies against TNF-α, p-HSL (Ser-563 and Ser-660; corresponding to human Ser-552 and Ser-650), total HSL, tubulin were purchased from Cell Signalling Technologies (Beverly, MA) and antibodies against PLIN1 and β-actin were purchased from Progen (Heidelberg, Germany) and Sigma-Aldrich, respectively. Secondary antibodies IgG-HRP (IgG conjugated to horse radish peroxidase) were purchased from Sigma-Aldrich. Antibody-antigen complexes were detected by chemiluminescence using ECL™ Select Western Blotting Detection Kit (GE Healthcare) as indicated in manufacturer’s instructions.

### Statistical Analyses

Data displayed in figures and tables are mean ± standard error of the mean (SEM). When appropriate, the data was ^10^log-transformed to become normally distributed. Results were analysed by unpaired Student t-test. In miR-145 over-expression experiments, relative values of glycerol, TNF-α secretion and mRNA expression were corrected by transfection efficiency and analysed by linear regression.

## Results

### Screening of microRNAs Identifies TNF-α as One Mediator of miRNA Effects on Human Adipocyte Lipolysis

**Figure 1:**
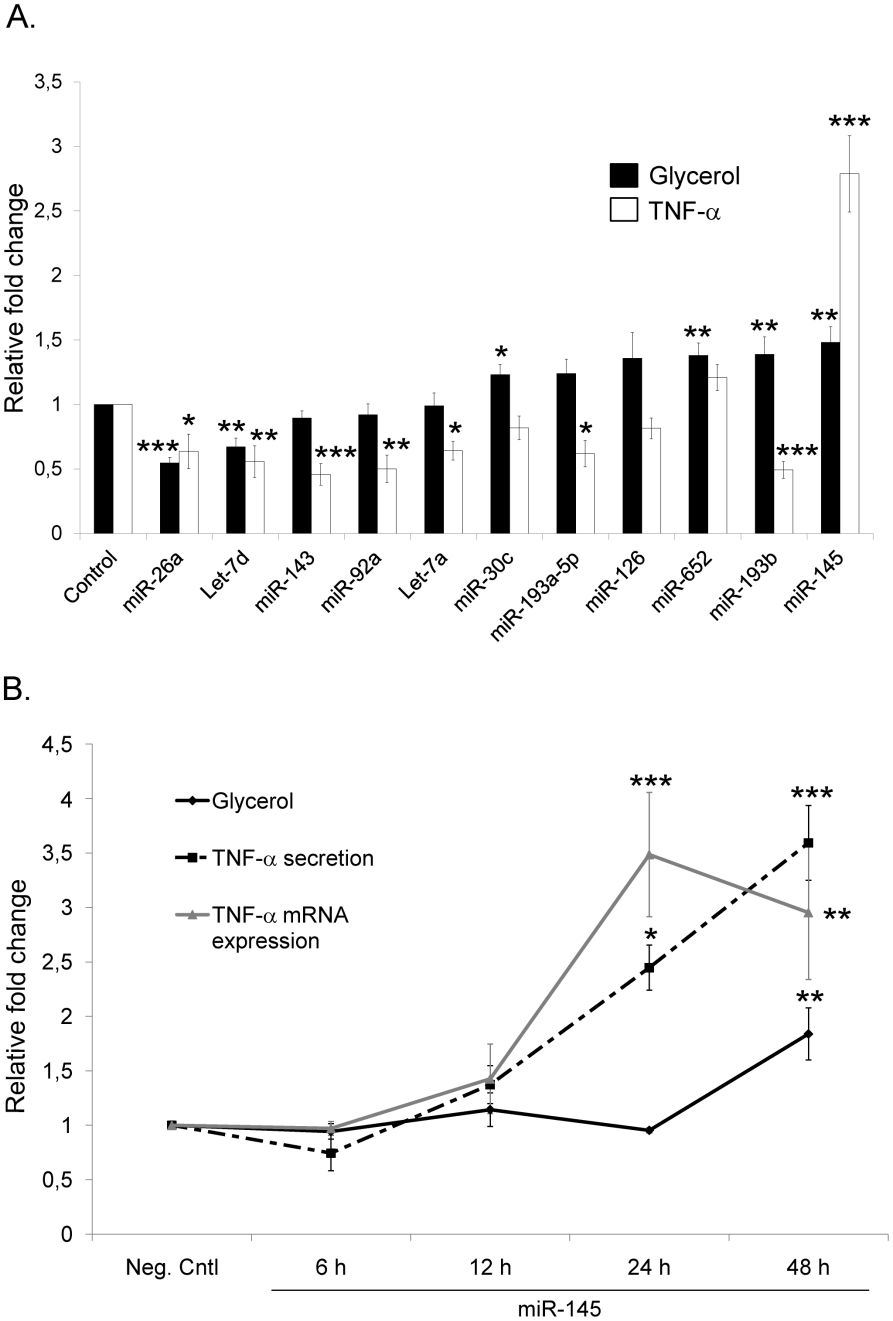
Transfection screening of microRNAs identifies TNF-α as a key mediator of miRNA effects on human adipocyte lipolysis. (A) Human differentiated adipocytes were transfected with each individual miRNA Mimics or Negative Control (40 nM) for 48 h as described in experimental procedures. After 48 h, conditioned media was collected and glycerol release and TNF-α secretion were measured. Glycerol levels were evaluated by a bioluminescence method in three to six biological/independent experiments. TNF-α secretion values were detected by ELISA in at least, two biological/independent experiments. Values are shown as mean ± SEM and expressed as relative fold change *vs*. Neg. Cntl. Statistical differences were analyzed by Student t-test comparing Mimics Neg. Cntl *vs*. Mimics of each miRNA: *p<0.05; **p<0.01; ***p<0.001. (B) MiR-145 was over-expressed in human differentiated adipocytes in a time-course experiment. Conditioned medium was collected at selected time-points post-transfection (6 h –12 h –24 h –48 h) for determination of glycerol (black line) and TNF-α secretion (black-broken line) levels. Cells were harvested for RNA and TNF-α mRNA expression (grey line) levels were determined. Results are indicative of three biological/independent experiments. Transfection efficiency showed ∼2×10^4^ fold-change up-regulation of individual miR-145 as compared to control (see Figure S1). Values are shown as mean ± SEM and expressed as relative fold change *vs*. Neg. Cntl. of each time-point. Statistical differences were analyzed by Student t-test comparing Mimics miR-145 *vs*. corresponding Neg. Cntl at each indicated time-point: *p<0.05; **p<0.01; ***p<0.001.

In order to study whether microRNAs have an impact on adipocyte lipolysis, we selected a set of eleven miRNAs that we have previously described/identified as considerably and abundantly expressed in human WAT and isolated fat cells of a large number of subjects [18]. We performed a transfection screen where individual miRNAs were over-expressed in human *in vitro* differentiated adipocytes followed by measures of glycerol release (an indicator of lipolysis). While five out of the eleven miRNAs had no effect on adipocyte lipolysis, four (miR-30c, -652, -193b, -145) significantly increased and two (miR-26a and let-7d) significantly decreased glycerol release (Figure 1A).

We also determined TNF-α protein levels in the same samples (Figure 1A). Seven miRNAs (miR-26a, let-7d, -143, -92a, let-7a, -193a-5p, -193b) significantly decreased while only one (miR-145) significantly increased TNF-α levels in the conditioned media. The TNF-α concentrations in the conditioned media varied between 0.2 pg/mL (for miR-26a) and 1.1±0.2 pg/mL (for miR-145). For three miRNAs, the effects on glycerol and TNF-α release were parallel, i.e. both measures were either decreased (miR-26a and let-7d) or increased (miR-145). Since it is more feasible to determine mechanisms underlying increased (in contrast to attenuated) basal lipolysis, further studies were focused on miR-145.

To determine the temporal order of miR-145 effects on lipolysis rate and TNF-α production, we performed a time-course experiment where miR-145 was over-expressed and TNF-α mRNA/protein secretion and glycerol release were measured at several time-points (6, 12, 24 and 48 h) post-transfection (Figure 1B). TNF-α mRNA expression levels started to increase at 12 h and reached a *plateau* at 24 h (p<0.001). This was followed by a time-dependent increase in TNF-α secretion from 12 h, 24 h (p<0.05) to 48 h (p<0.001). These changes preceded the effects observed on lipolysis since glycerol levels remained unchanged at early time points (6 h –12 h –24 h) and were significantly increased only after 48 h (p<0.001). MiR-145 over-expression levels were similar at all studied time-points (see Figure S1). This suggests that the effects of miR-145 on adipocyte lipolysis are preceded by an increased production of TNF-α and that the quantitative effects on glycerol release correlate with TNF-α levels.

### MiR-145 Alters TNF-α Signaling and Induces Tightly Correlated Changes in Lipolysis and TNF-α

**Figure 2:**
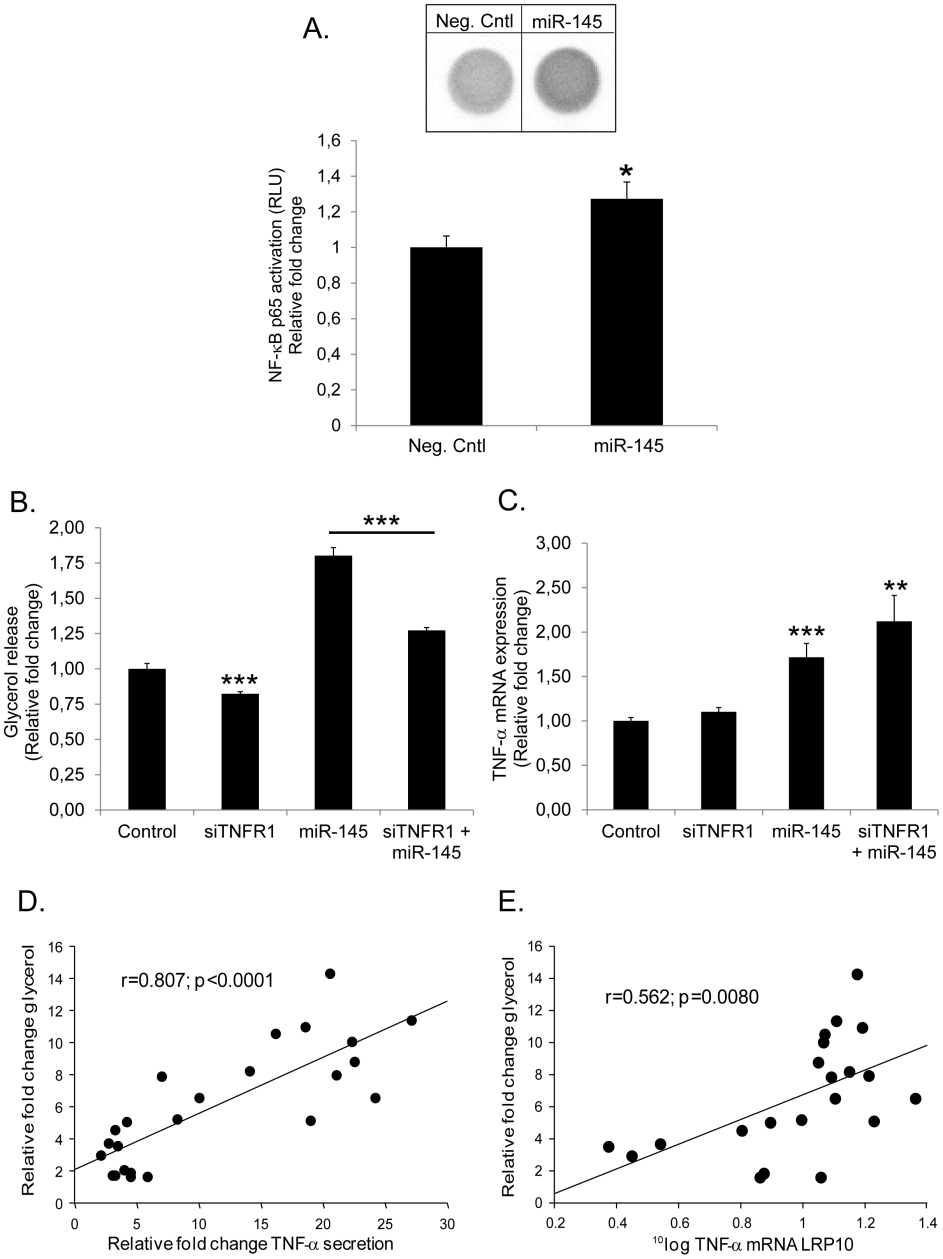
miR-145 alters TNF-α signaling and induces tightly correlated changes in lipolysis and TNF-α. (A) Human differentiated adipocytes were transfected with mimics miR-145 or Neg. Cntl (40 nM) for 15 h. After incubation time (15 h), nuclei were isolated and 2.5 µg of nuclear extract was used to perform p65 transactivation assay as described by manufacturer. Figure depicts representative wells of Neg. Cntl and miR-145-treated adipocytes and its relative quantification graph of three biological/independent experiments. Values are shown as mean ± SEM and expressed as relative fold change *vs*. Neg. Cntl. Statistical differences were analyzed by Student t-test: *p<0.05. (B) TNF-α receptor 1 (TNFR1) was silenced with siRNA for 24 h prior to co-transfection with miRNA Mimics (Neg. Cntl/miR-145) for additional 48 h in human differentiated adipocytes. After incubation time, conditioned medium was collected to measure glycerol release and cells were harvested for (C) TNF-α mRNA measurements. Results are representative of three biological/independent experiments. Values are shown as mean ± SEM. Statistical differences (*vs*. Neg. Cntl) were analyzed by Student t-test: **p<0.01; ***p<0.001. (D) Correlation between glycerol *vs*. TNF-α secretion (measured in conditioned medium/supernatant) and *vs*. (E) TNF-α mRNA levels in miR-145-transfected adipocytes *in vitro* for 48 h. Values are expressed as relative fold change *vs*. Neg. Cntl. (in D and E) after correction by transfection efficiency and were compared by linear regression. Each dot represents a technical replicate from 7–8 biological/independent experiments.

NF-κB pathway is an important regulator for TNF-α-induced lipolysis in human adipocytes [6]. We evaluated whether miR-145-induced changes of TNF-α (mRNA and protein) were related to increased transcriptional activity of the NF-κB complex by assessing nuclear translocation of p65 following miR-145 over-expression. A significant ∼30% increase in p65 translocation was observed 15 h after transfection of miR-145 mimics (Figure 2A). As expected from the time-course data in Figure 1B, glycerol levels remained unchanged at this early time-point (values not shown).

The results in Figures 1–2 indicate that the ability of miR-145 to stimulate adipocyte lipolysis, may be secondary to increased TNF-α production. Therefore, we next wanted to evaluate if the increase on adipocyte lipolysis after miR-145 treatment was directly mediated via the TNF-α signaling pathway. To test this hypothesis, we attenuated the expression of human fat cell TNFR1, the primary receptor mediating the lipolytic effect of TNF-α in human fat cells [3], alone or in combination with miR-145 over-expression, and assessed effects on basal lipolysis. We could confirm that selective TNFR1 knock-down in human fat cells (∼90% down-regulation, p<0.001, Figure S2) caused a significant decrease in basal glycerol levels (Figure 2B). Furthermore, TNFR1 down-regulation attenuated the effect of miR-145 on glycerol release. This was observed despite a significant and similar increase in TNF-α mRNA levels in miR-145 over-expressing cells transfected with or without TNFR1 siRNA (Figure 2C). Additional experiments demonstrated that there was a positive and highly significant correlation between glycerol release and the levels of TNF-α secretion (Figure 2D) or TNF-α mRNA (Figure 2E) in miR-145 overexpressing adipocytes at 48 h.

### ADAM17 is a miR-145 Target that Regulates TNF-α Processing in Human Adipocytes

**Figure 3:**
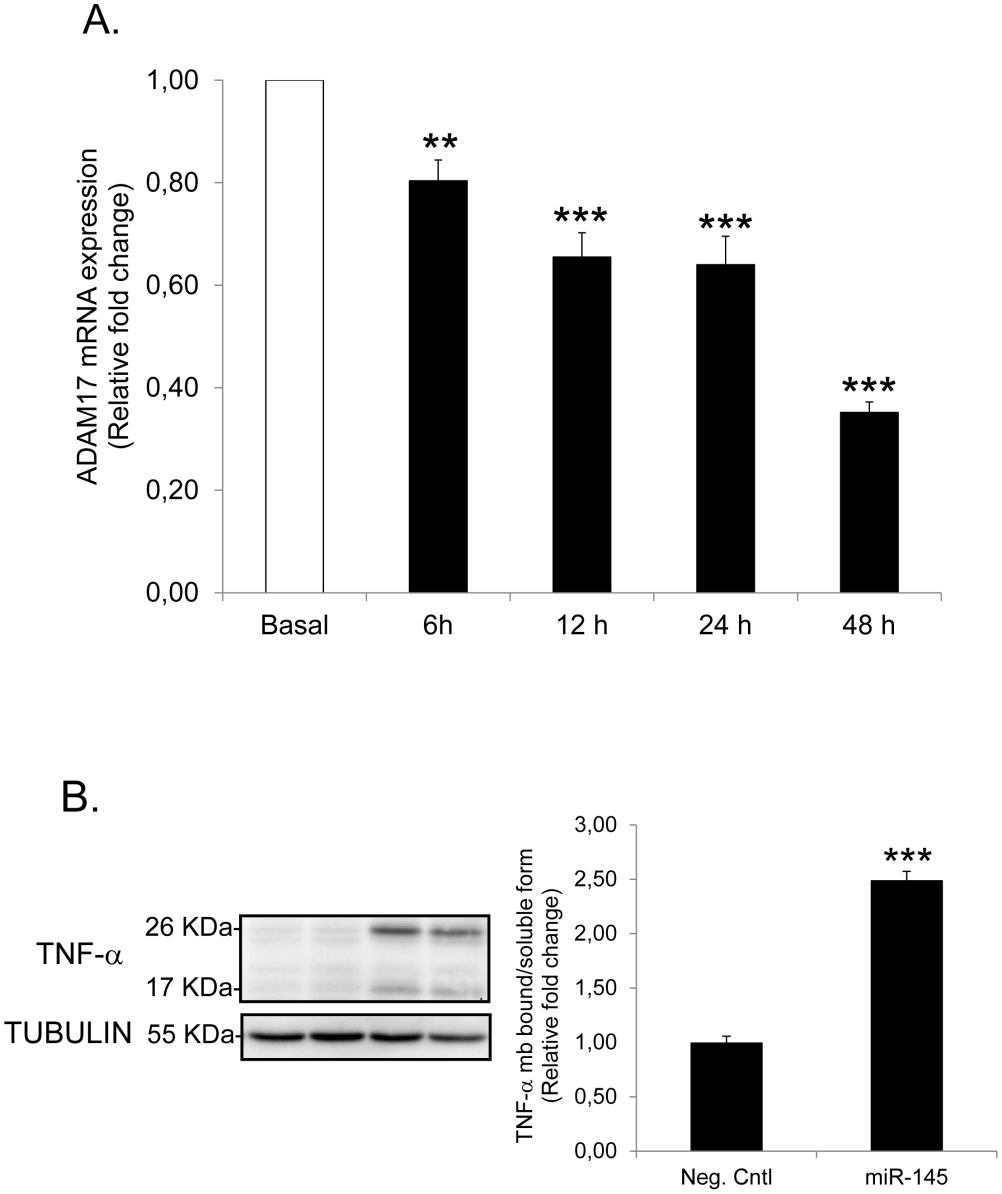
ADAM17 is a miR-145 target that regulates TNF-α processing in human adipocytes. (A) mRNA expression levels of ADAM17 after miR-145 over-expression at 6 h –12 h –24 h –48 h. Results presented are obtained from three biological/independent experiments. Values are shown as mean ± SEM and expressed as relative fold change *vs*. Neg. Cntl. at each corresponding time-point. (B) MiR-145 was over-expressed in human differentiated adipocytes for 48 h and whole cell lysates were analyzed by Western blot. TNF-α protein levels are expressed as ratio of TNF-α membrane bound (26 KDa) *vs*. soluble form (17 KDa). Results are representative of two biological/independent experiments. Values are shown as mean ± SEM. Statistical differences (*vs*. Neg. Cntl) were analyzed by Student t-test: **p<0.01; ***p<0.001.

Recent studies have shown that miR-145 binds to the 3′UTR of ADAM17 mRNA and thereby regulates its expression [21], [25]. Therefore, we investigated whether alterations in ADAM17 expression might be involved in the regulation of TNF-α secretion by miR-145. In a time-course experiment, ADAM17 mRNA was significantly down-regulated by miR-145 in a temporal manner (Figure 3A) while over-expression of miR-145 lead to a marked increase in the ratio of membrane-bound (26 KDa) *vs*. the soluble form (17 KDa) of TNF-α at 48 h (Figure 3B).

### MiR-145 Regulates HSL (but not PLIN1) Phosphorylation and PDE3B mRNA Expression

**Figure 4:**
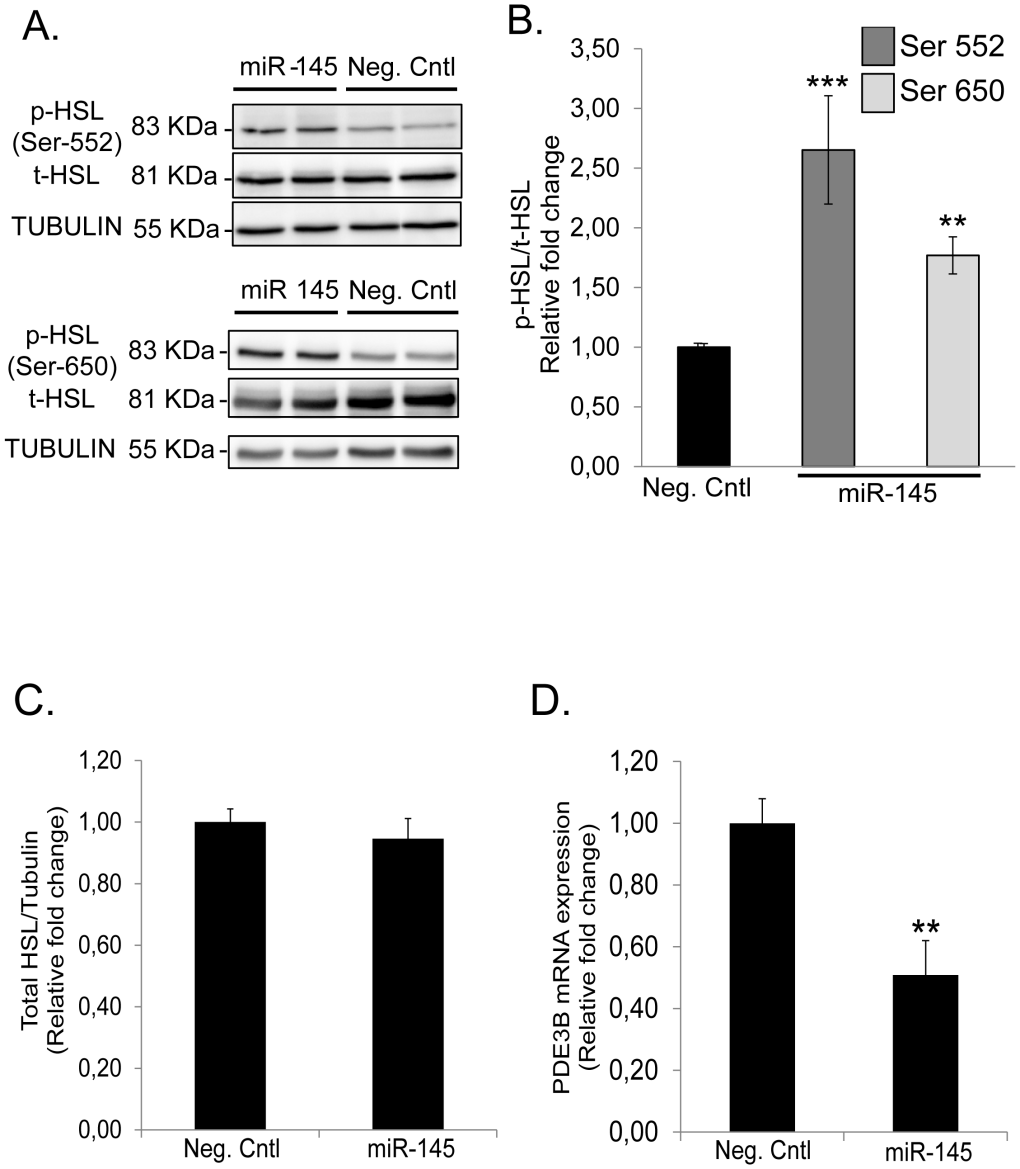
miR-145 increases HSL phosphorylation at activating residues (but does not change total protein content of HSL) and down-regulates PDE3B. (A) Representative blots of protein expression levels of phosphorylated HSL (Ser-552 and Ser-650) and total HSL in human differentiated adipocytes transfected with miR-145 mimics for 48 h. (B) Relative quantification by densitometry of above depicted blots for p-HSL (Ser-552, dark grey; Ser-650, light grey) and (C) total HSL. Equal amounts of total protein were loaded and separated by SDS-PAGE as indicated in experimental procedures. Total HSL levels were corrected by tubulin expression and p-HSL levels were corrected by total HSL protein content. Values are shown as mean of pooling results from three biological/independent experiments. (D) Relative expression of PDE3B after miR-145 over-expression in human differentiated adipocytes for 48 h. Results are representative of four biological/independent experiments. Values are shown as mean ± SEM. Statistical differences (*vs*. Neg. Cntl) were analyzed by Student t-test: **p<0.01; ***p<0.001.

**Figure 5:**
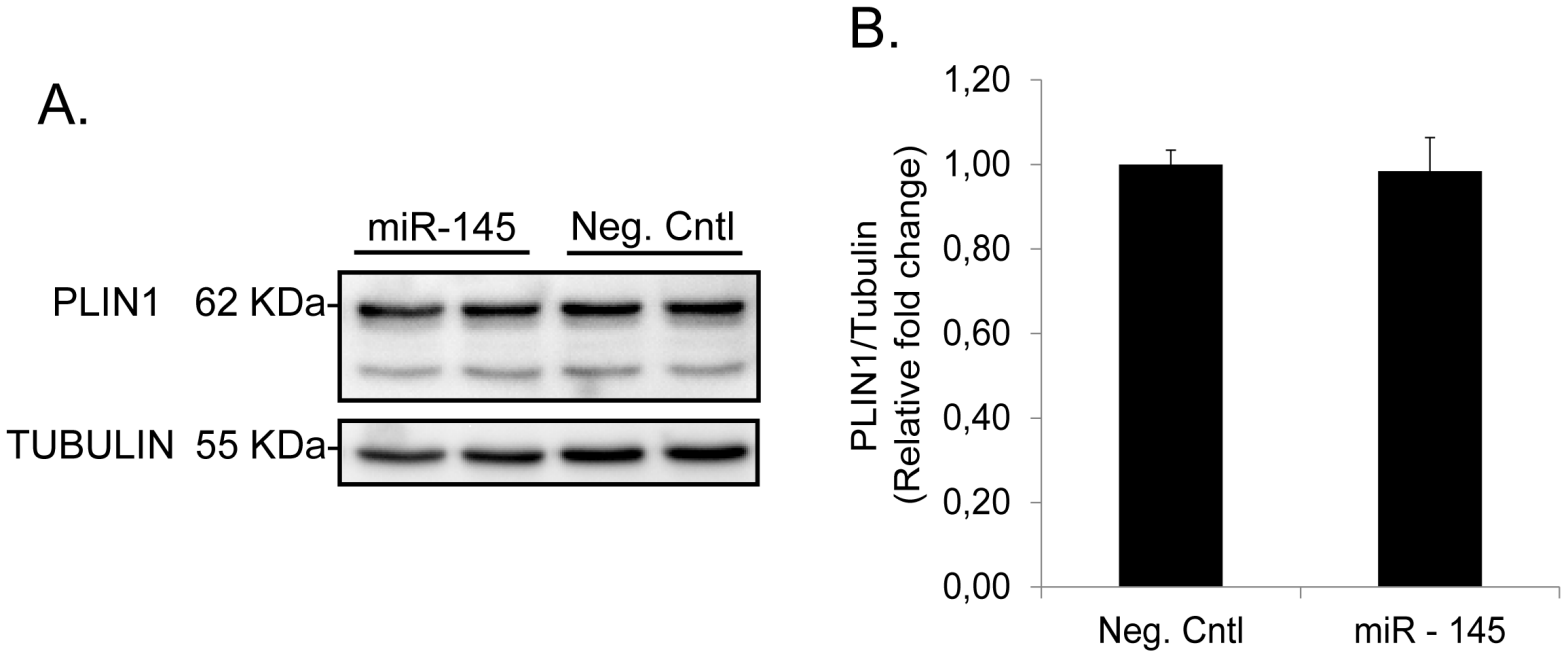
miR-145 does not affect phosphorylation and protein content of PLIN1. (A) Representative blot of total protein content of PLIN1 after miR-145 over-expression in human differentiated adipocytes for 48 h. PLIN1 protein content was corrected by tubulin as described in experimental procedures. (B) Relative quantification of PLIN1 *vs*. tubulin (n = 3).

It is well-established that TNF-α increases the phosphorylation of HSL and PLIN1 [3], [4], [9], [26] and decreases mRNA expression of PDE3B [9]. Over-expression of miR-145 for 48 h induced a significant increase in phosphorylation of HSL at activating residues (Ser-552 and Ser-650) (Figure 4A and 4B) but did not alter total HSL protein levels (Figure 4C). We also measured PDE3B mRNA expression at different time points up to 48 h after transfection with miR-145 mimics (Figure 4D). There was a significant down-regulation in gene expression first observable at 24 h post-transfection (Figure S3). In contrast, we did not observe any effects on PLIN1 phosphorylation status as miR-145 did not induce any shift in the protein bands towards a higher molecular weight. In addition, no significant changes were observed in total PLIN1 protein content (Figure 5A and 5B).

## Discussion

The present work demonstrates that miRNAs regulate adipocyte lipolysis adding this metabolic action to the pleiotropic effects of this class of molecules. We found that three (miR-26a, let-7d and miR-145) of the eleven tested miRNAs had a concordant effect on TNF-α secretion and glycerol release. This suggests involvement of TNF-α in their lipolytic effect although we cannot exclude TNF-α-independent mechanisms as well. Importantly, the concentration of TNF-α in conditioned media from miR-26a, let-7d and miR-145 overexpressing cells varied between 0.2 pg/mL (for miR-26a) and 1.1 pg/mL (for miR-145) which is within the physiological concentration interval of TNF-α in the interstitial space of human adipose tissue [27], [28]. This suggests that small variations in local TNF-α concentrations lead to clearly detectable changes in adipocyte lipolysis. On average, the pro-lipolytic effects of miR-145 resulted in an approximate 50% increase in glycerol levels in transfection screening (Figure 1).

In the over-expression experiments, miRNAs were >100 fold up-regulated. At this high-level, there might be off-target effects. This is generally a methodological issue in miRNA over-expression experiments. However, we always compare cells treated with specific mimics miRNA with mimics Negative Control. Furthermore, in a recent study [18], we found no changes in fat cell morphology or function due to specific miRNA over-expression.

In order to study the axis miRNA-TNF-α lipolysis more in detail, we selected miR-145 because it was the only miRNA that stimulated both TNF-α and glycerol release. Several lines of evidence suggest that miR-145 stimulates lipolysis by increasing the endogenous production of TNF-α. First, there was a strong correlation between changes in TNF-α mRNA/TNF-α secretion and lipolysis following miRNA over-expression. Second, TNFR1 knock down abrogates miR-145-induced lipolysis suggesting a causal relationship between the effects of miR-145 and TNFR1-mediated signaling. Importantly, the finding that TNFR1 depletion in fat cells attenuates lipolysis suggests that endogenous TNF-α production is important for basal adipocyte lipolysis. Third, miR-145-induced effects on adipocyte lipolysis occurred after induction of TNF-α gene and protein secretion. Fourth, NF-κB, which stimulates TNF-α transcription in an auto−/paracrine loop, was activated prior to the peak in TNF-α secretion/mRNA expression following miR-145 transfection. This indicates that NF-κB induction may be a mechanism through which miR-145 regulates TNF-α expression. Moreover, since TNF-α induction by miR-145 was not dependent on TNFR1 expression, it is plausible that the TNF-α/NF-κB feedback loop might be mediated via TNFR2 while TNF-α-induced lipolysis is primarily induced via TNFR1. Nevertheless, the direct link between miR-145 and NF-κB is not clear at the moment. Since miRNAs are down-regulators of gene expression, there are probably several steps up-stream of NF-κB activation linking miR-145 to TNF-α production which could include one or more negative regulators of NF-κB. However, to identify this/these candidates might prove difficult and was out of the scope of this study.

TNF-α can either be present in soluble or a cell membrane-bound state. Although both forms are biologically active, membrane-bound TNF-α is thought to be responsible for cell-to-cell interaction and activate TNF-α signaling pathways in adjacent cells thereby promoting the development of an intense inflammatory response within the cellular micro-environment [29]. Interestingly, it has recently been shown in human non-adipose cells (renal and hepatic cancer) that miR-145 decreases ADAM17 expression by direct binding to the 3′UTR of the gene [21], [25]. Although this interaction was not assessed in human adipocytes, the fact that both cell systems were human, suggests that a similar interaction may be present in other human cells including fat cells. The observations that miR-145 over-expression resulted in a time-dependent decrease in ADAM17 mRNA expression as well as a significant increase in the ratio of membrane-bound/soluble TNF-α, suggests that miR-145 may increase TNF-α activity in human adipocytes, at least in part, through inhibition of ADAM17. This fact further emphasizes that the increase in TNF-α production by miR-145 is not only mediated by a direct effects on TNF-α transcription but also probably through an alteration of pre-existing levels within the cell (e.g. TNF-α processing). In a broader context, this would mean that adipocytes become more “sensitive” to certain pre-existing factors (such as TNF-α) and therefore, the regulatory system develops and amplifies an inflammatory response at the intra- and extracellular level, mediated in an auto−/paracrine manner by TNF-α. This finding would support the hypothesis that the effect of miR-145 on lipolysis is complex, involving several intermediates and complementary interactions between them. However, we cannot exclude the possibility that other genes targeted by miR-145 might contribute to regulate TNF-α production as well and/or that miR-145 may also exert TNF-α-independent effects on lipolysis.

Final steps in TNF-α-mediated lipolysis activation include HSL [30] and PLIN1 phosphorylation [9]. Our results suggest that HSL but not PLIN1 phosphorylation is involved in miR-145 stimulated lipolysis through TNF-α at least under the conditions and time-points (48 h) used in this study. It could be speculated that phosphorylation of HSL might constitute an earlier event than PLIN1 phosphorylation. Indeed, we cannot rule out that PLIN1 phosphorylation could be observed at later time-points although total protein content remained unaltered. Finally, the inhibition of PDE3B gene expression might constitute an additional mechanism through which miR-145 affects adipocyte lipolysis. However, this effect seems to be secondary to TNF-α expression rather than a direct miRNA-mediated effect, since PDE3B down-regulation occurred at later time-points (24 h and 48 h) after miR-145 over-expression.

Our screen included eleven miRNAs out of which only three displayed concordant effects on lipolysis and TNF-α production. For the remaining candidates, a few attenuated TNF-α without altering lipolysis (miR-143, -92a and -193a-5p). This could possibly depend on the fact that it is sometimes difficult to assess reductions (compared with increases) in glycerol levels. It is therefore possible that increasing the number of experiments would identify (small) reductions in glycerol and TNF-α release also with these miRNAs. Moreover, a few miRNAs increased lipolysis without significant changes in TNF-α levels (miR-30c, -652) or even with a reduction in its release (miR-193b). These data suggest that specific miRNAs may alter lipolysis via TNF-α-independent mechanisms. However, investigating these alternative pathways was out of the scope of this present study.

On the basis of the present findings, we propose that TNF-α production and lipolysis can be regulated by miRNAs. MicroRNA-145 enhances the production/release of TNF-α and increases the amount of membrane bound TNF-α via inhibition of ADAM17. It also activates NF-κB, decreases PDE3B expression and enhances HSL phosphorylation. All these events promote lipolysis activation and taken together, suggest that the effects of miR-145 on lipolysis are, at least in part, mediated by the effects on TNF-α.

If the present findings are relevant for other species, remains to be established. Future studies are also needed to elucidate the mechanisms mediating the effects of the other miRNAs identified in this study.

## Supporting Information

Figure S1 **Quantification of over-expression of miR-145 in human differentiated pre-adipocytes.** Human differentiated adipocytes were transfected with miR-145 Mimics and collected at several time-points post-transfection (6 h –12 h –24 h –48 h) as described in material and methods. Cells were harvested for RNA and relative miR-145 expression levels were determined. Results are indicative of three biological/independent experiments. Values are shown as mean ± SEM and expressed as relative fold change *vs*. Neg. Cntl. of each time-point.
(TIF)

Figure S2 **Quantification of TNFR1 mRNA levels after specific gene silencing with siRNA.** TNFR1 was silenced with siRNA in human differentiated adipocytes as described in material and methods. Cells were harvested for RNA and relative TNFR1 mRNA expression levels were determined. Results are indicative of three biological/independent experiments. Values are shown as mean ± SEM. Statistical differences were analyzed by Student t-test: ***p<0.001.
(TIF)

Figure S3 **Quantification of PDE3B mRNA levels miR-145 over-expression time-course.** MiR-145 was over-expressed in human differentiated adipocytes for 6 h –12 h –24 h –48 h. Cells were harvested for RNA and PDE3B mRNA expression levels were determined. Results are indicative of three biological/independent experiments. Values are shown as mean ± SEM. Statistical differences were analyzed by Student t-test: **p<0.01.
(TIF)
