# Supplementary figures and images for: MicroRNAs Regulate Human Adipocyte Lipolysis: Effects of miR-145 Are Linked to TNF-α
Source: PLoS One. 2014 Jan 24;9(1):e86800. doi: 10.1371/journal.pone.0086800 (PMC3901697; doi:10.1371/journal.pone.0086800)

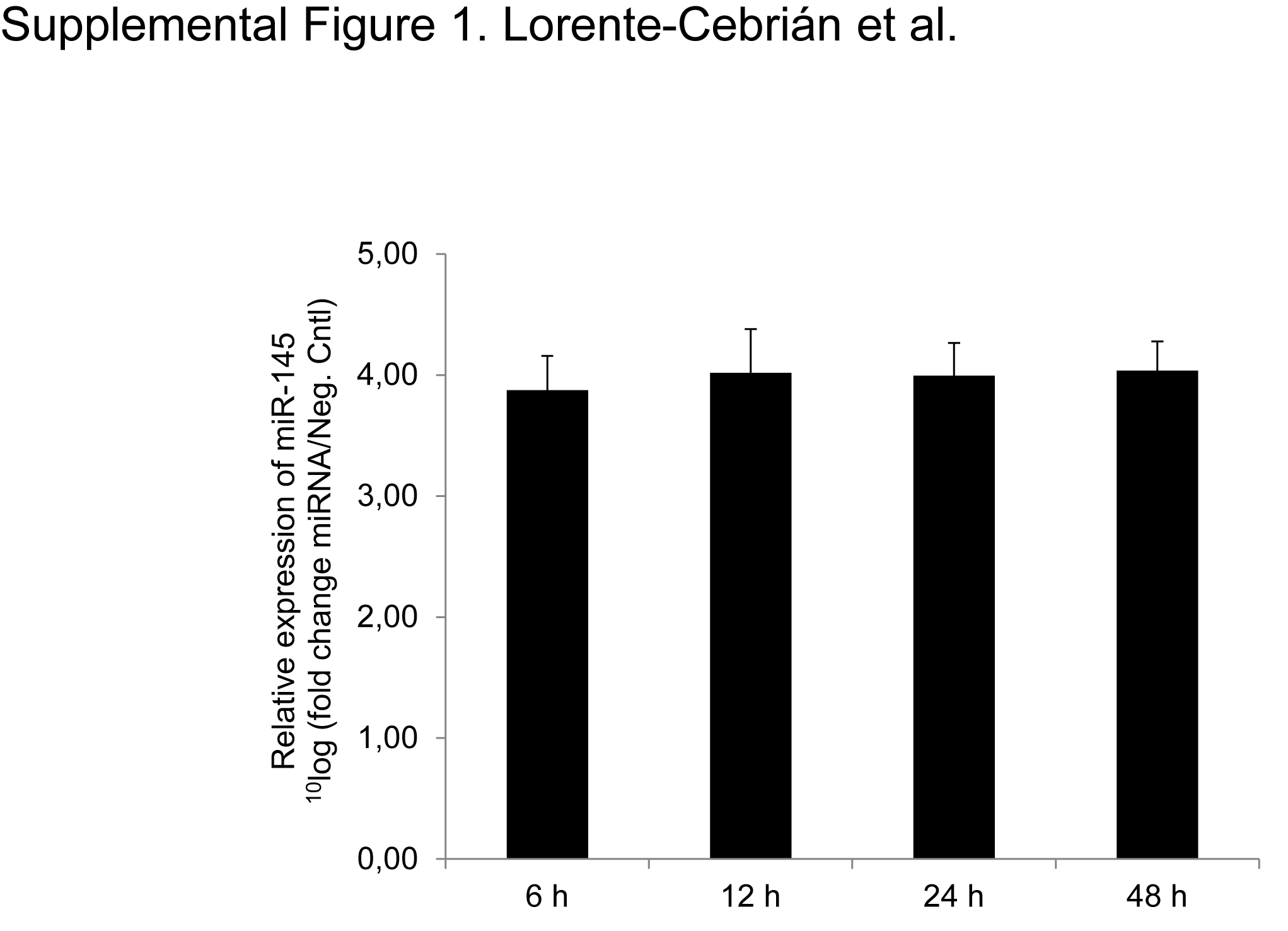

Supplement: Figure S1 — Quantification of over-expression of miR-145 in human differentiated pre-adipocytes. Human differentiated adipocytes were transfected with miR-145 Mimics and collected at several time-points post-transfection (6 h –12 h –24 h –48 h) as described in material and methods. Cells were harvested for RNA and relative miR-145 expression levels were determined. Results are indicative of three biological/independent experiments. Values are shown as mean ± SEM and expressed as relative fold change vs. Neg. Cntl. of each time-point. (TIF) [file pone.0086800.s001.tif]

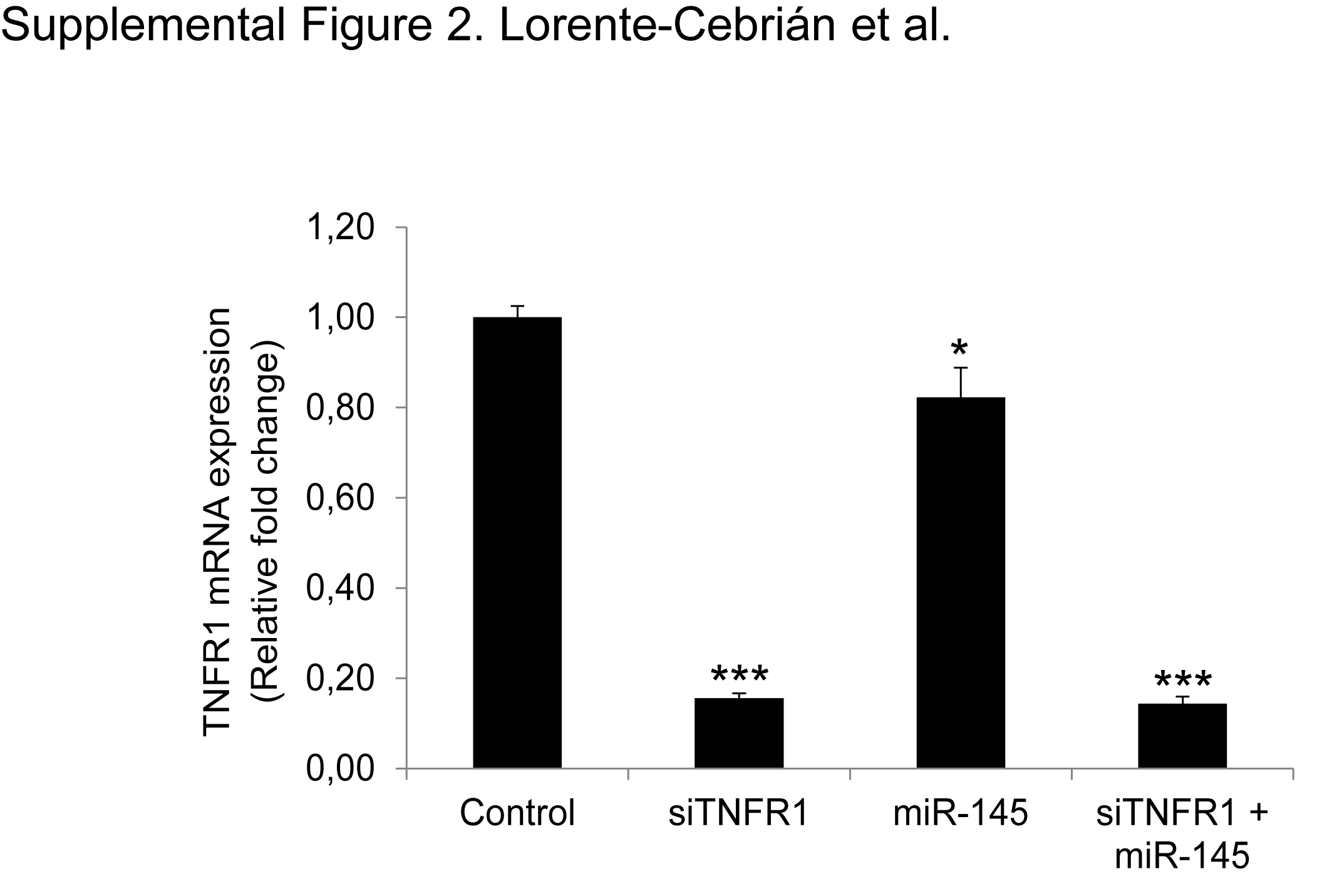

Supplement: Figure S2 — Quantification of TNFR1 mRNA levels after specific gene silencing with siRNA. TNFR1 was silenced with siRNA in human differentiated adipocytes as described in material and methods. Cells were harvested for RNA and relative TNFR1 mRNA expression levels were determined. Results are indicative of three biological/independent experiments. Values are shown as mean ± SEM. Statistical differences were analyzed by Student t-test: ***p<0.001. (TIF) [file pone.0086800.s002.tif]

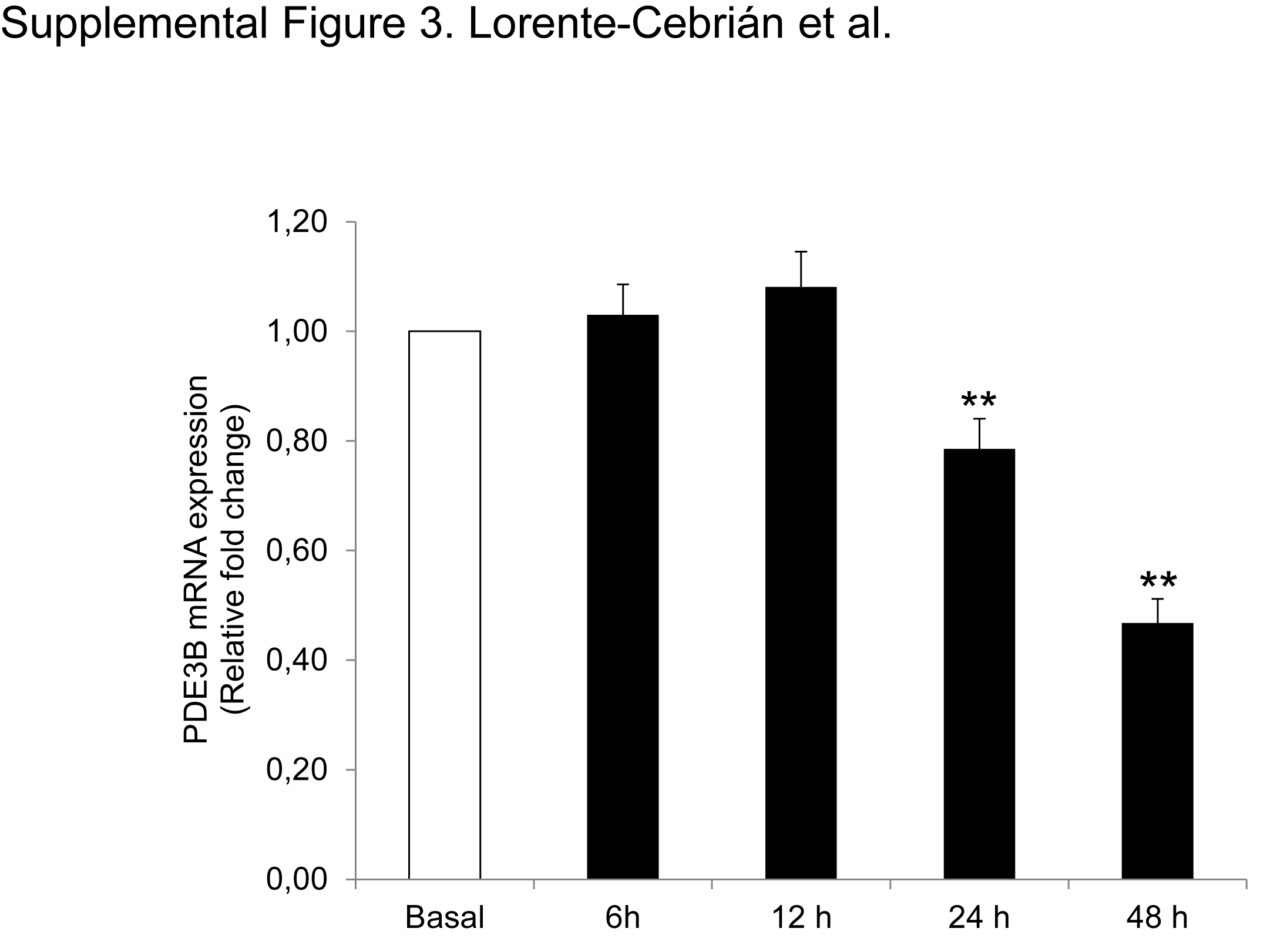

Supplement: Figure S3 — Quantification of PDE3B mRNA levels miR-145 over-expression time-course. MiR-145 was over-expressed in human differentiated adipocytes for 6 h –12 h –24 h –48 h. Cells were harvested for RNA and PDE3B mRNA expression levels were determined. Results are indicative of three biological/independent experiments. Values are shown as mean ± SEM. Statistical differences were analyzed by Student t-test: **p<0.01. (TIF) [file pone.0086800.s003.tif]
